# Advancing Global Hepatitis B Elimination: The Case for Using Maize as a Low-Cost, Heat-Stable, and Scalable Oral Vaccine

**DOI:** 10.3390/vaccines14070578

**Published:** 2026-06-30

**Authors:** Muneaki Watanabe, John A. Howard

**Affiliations:** Applied Biotechnology Institute Inc., San Luis Obispo, CA 93407, USA; mwatanabe@appliedbiotech.org

**Keywords:** hepatitis B, oral boost, oral vaccines, transgenic maize, maize germ, mucosal immunity, thermostability, plant-based vaccines, VLP, HBsAg, vaccine accessibility, bioencapsulation, cold-chain independent

## Abstract

Because hepatitis B virus (HBV) remains a major global health burden, innovative strategies are essential to achieve the World Health Organization’s goal of eliminating viral hepatitis and closing persistent coverage gaps for injectable vaccines. While parenteral administration remains the gold standard for immunization, constraints such as cold-chain dependence and needle-associated barriers limit its reach, particularly in resource-constrained environments. This review summarizes work aimed at a plant-produced orally delivered vaccine as a transformative, scalable step towards global hepatitis B elimination. Early studies demonstrated proof of concept for the oral delivery of plant-produced hepatitis B vaccine candidates, including human trials using lettuce and potato as the host, but they were limited by low antigen yields and instability. In contrast, maize-produced antigens represent a significant advancement, achieving high levels of accumulation and utilizing the seed’s natural desiccation physiology for bioencapsulation to protect the antigen from digestion in the gastrointestinal tract. Mechanistically, this platform enables timed antigen release in the duodenum, promoting M-cell uptake and CD103^+^ (cells expressing CD103 known as integrin alpha E) dendritic cell (DC) presentation, thus encouraging immunogenic programming over oral tolerance. In addition, defatting the grain by supercritical fluid extraction further improves antigen thermostability up to 45 °C for one month and ambient temperatures for one year, maintaining structural integrity under extreme conditions in accordance with the International Council for Harmonization of Technical Requirements for Pharmaceuticals for Human Use (ICH) stability guidelines. Current recommendations for immunization are for three parenteral administrations using the hepatitis B surface antigen (HBsAg). The primary dose is usually given shortly after birth as a part of a multivalent vaccine. Therefore, initial studies for the oral plant-based vaccine have focused on using an oral boost after the parenteral prime. Data to support this premise are summarized along with co-administration of an oral and parental administration to elicit a stronger immune response. By overcoming past issues related to dose density and stability, this scalable, needle-free platform offers a practical way to eliminate global hepatitis B virus (HBV) transmission, especially in resource-constrained environments.

## 1. Introduction

Hepatitis B virus (HBV) remains a significant global and domestic public health challenge despite the availability of highly effective recombinant vaccines [1,2,3,4,5,6,7,8,9,10,11] for more than four decades. Achieving and maintaining protective immunity is essential to controlling infection and preventing the long-term consequences of chronic HBV, including cirrhosis and hepatocellular carcinoma [12,13,14,15,16]. Universal infant vaccination programs have demonstrated remarkable success [2,6,12,17,18,19] in countries with high coverage and HBV incidence has fallen sharply among infants and young children. In the United States, routine childhood immunization has resulted in a >96% reduction in HBV cases in these age groups [1,2,5,7,12,20,21,22]. However, adults continue to account for the majority of new infections [1,5,13,22,23,24,25]. Many remain unvaccinated or incompletely vaccinated, leaving them vulnerable to exposure through well-documented transmission routes such as sexual contact, unsafe injection practices, injection-drug use, and accidental bloodborne exposure [1,12,20,22,23,24] in healthcare and community settings. These behavioral and occupational risk factors mean that, even in regions with strong pediatric vaccine programs, millions of adults remain susceptible [1,2,5,7,12,20,21,22,23,24].

The persistence of adult HBV transmission highlights an important gap between vaccine availability and real-world immunity [1,5,7,12,20,21,22,23,24]. Injectable HBV vaccines are clinically effective but operationally burdensome: they require cold-chain storage, skilled personnel for administration, sterile needles, and adherence to multi-dose schedules [2,5,12,13,20,23,24,26]. These requirements create logistical and behavioral barriers that reduce coverage, not only in low-resource settings but also within high-income countries such as the United States, where missed opportunities, limited healthcare access, vaccine hesitancy, and needle-related aversion continue to limit adult immunization rates. Addressing these constraints will require complementary delivery approaches that can overcome logistical obstacles and broaden access to populations at ongoing risk.

Oral vaccination presents a compelling alternative with the potential to meet these needs. By engaging the mucosal immune system (the primary site of exposure for 90% of pathogens) oral vaccines can elicit both mucosal and systemic immunity [3,27,28,29,30] while enabling the possibility of needle-free, self-administered dosing. These immunological and practical advantages support their potential role in adult catch-up vaccination, booster immunization, and community-based disease prevention. However, the successful development of oral vaccines depends heavily on the production system used to generate the antigen, as oral delivery places unique demands on antigen concentration in the host tissue, stability, encapsulation, and scalability [3,17,27,28,29,31,32,33,34,35,36,37,38,39,40,41,42,43,44,45].

Advances in biotechnology have expanded the range of platforms capable of producing HBsAg for oral delivery, including microbial systems such as *Escherichia coli* and yeast, and oral plant-based systems such as rice, potato, lettuce, and maize [3,28,30,33,34,36,38,41,45,46,47,48,49,50,51,52,53]. Each system offers distinct advantages and limitations related to yield, stability, cost, and regulatory feasibility. Among plant systems, seed-based platforms stand out for their natural ability to stabilize antigens, and in particular, maize has shown promising levels of antigen accumulation and thermostability [32,38,39,40,41,44,54,55]. These characteristics position maize as a candidate with strong translational potential, though its role must be evaluated within the broader landscape of both traditional and alternative vaccine platforms.

This paper provides a comprehensive narrative overview of the existing maize-derived vaccine literature and therefore aims to compare injectable and oral HBV vaccine modalities, assess the strengths and limitations of microbial and plant-based expression systems for oral HBsAg production, and contextualize the emerging role of maize as a practical and scalable platform for booster and supplemental immunization strategies. By linking transmission dynamics, immunological requirements, and production-system capabilities, this review highlights how plant-derived oral vaccines, especially maize-based formulations, may contribute to closing persistent immunity gaps and expanding global HBV prevention efforts.

## 2. Overview of Injectable HBV Vaccines

Injectable hepatitis B vaccines have served as the foundation of global HBV prevention programs since their introduction in the early 1980s [3,6,39]. These vaccines have typically been produced by recombinant expression of the surface protein for HBV, HBsAg, in *Saccharomyces cerevisiae* or mammalian cell lines, followed by purification and formulation for intramuscular administration [6,7,31]. Historically, the earliest vaccines developed against HBV were plasma-derived [56], which provided a foundation baseline for subsequent recombinant technologies. Decades of clinical use have demonstrated their safety, high immunogenicity, and effectiveness in preventing both acute infection and progression to chronic HBV disease. In healthy individuals, seroconversion rates can exceed 95% [24,25,57,58], and widespread implementation of birth-dose and childhood vaccination programs has led to dramatic reductions in HBV prevalence among infants and young children [5,12,13,20,22].

Injectable vaccines elicit a strong systemic immune response, particularly neutralizing anti-HBsAg antibodies that correlate with long-term protection [5,12,13,20,22]. Their immunological performance is well-supported by extensive clinical data, standardized dosing regimens, and established manufacturing processes. Organizations such as the World Health Organization and national immunization programs continue to recommend a three-dose schedule beginning at birth as the primary strategy for preventing HBV infection across populations [5,12,13,20].

Despite their proven effectiveness, injectable vaccines encounter several logistical, behavioral, and other challenges, such as genetic issues, that limit their real-world impact [5,20,23,56]. Administration requires trained personnel, sterile injection equipment, and strict cold-chain storage requirements [26,59] that can strain healthcare systems, especially in rural, remote, or resource-limited areas. Additionally, pain, needle anxiety, and resistance to injections contribute to poor adherence to multi-dose schedules [12,20,22,60,61]. These issues primarily affect adolescent and adult populations, many of whom remain unvaccinated or only partially vaccinated despite ongoing exposure risks from sexual contact, unsafe injections, injection drug use, or occupational hazards [23].

These operational and behavioral barriers highlight important gaps in achieving and maintaining population-level immunity, even when effective vaccines exist [12,22,23]. While injectable vaccines remain the clinical gold standard for primary HBV immunization, 300 million people are chronically infected worldwide with nearly 1 million fatalities per year. The practical limitations of current vaccines underscore the need for complementary vaccination strategies that could expand reach, improve compliance, and reduce logistical burdens.

## 3. Oral Vaccine Delivery: Concept and Mechanism

Oral vaccination leverages the mucosal immune system, which serves as the body’s primary interface with ingested and inhaled antigens. Unlike injectable vaccines that predominantly induce systemic IgG responses, oral vaccines can stimulate both mucosal IgA and systemic immunity [3,4,17,38,39,45,46,62]. This dual response includes the gut-associated lymphoid tissue (GALT), where specialized microfold (M) cells shuttle luminal antigens to underlying DCs and macrophages [3,17,29,39,46]. In addition, antigens presented in the oral cavity can elicit mucosal and systemic responses, primarily the sublingual (under the tongue) mucosa, buccal (inner cheek) mucosa, and Waldeyer’s tonsillar ring (palatine/lingual tonsils). These antigen-presenting cells activate downstream adaptive responses, enabling coordinated local and systemic protection.

A key feature of the mucosal immune system is its ability to discriminate between harmful and harmless antigens. This protective filtering mechanism creates both opportunities and challenges for oral vaccines. When antigens are delivered in a particulate, pathogen-like form or accompanied by innate-stimulatory signals, uptake by M cells and immunogenic DC subsets promotes robust IgA and IgG responses [3,4,17,39,46]. In contrast, soluble antigens delivered with repetitive low concentrations, or without appropriate “danger” cues, are more likely to be processed by tolerogenic DCs that induce regulatory T cells and suppress effector immunity [3,29,63,64,65]. As a result, the immunological context of antigen delivery (dose, physical form, stability, and presentation) strongly influences whether oral exposure results in active immunity or oral tolerance. Effective oral vaccine design must therefore ensure antigen survival, particulate structure, and/or adjuvant signals that favor immunogenic rather than tolerogenic pathways [29,48,66].

A major biological challenge is the survival of antigens in the gastrointestinal tract. Gastric acidity, proteolytic enzymes, bile salts, and mechanical digestion can denature or fragment recombinant proteins, including HBsAg, before they reach GALT. Various strategies have been used to enhance antigen survival, such as encapsulation in polymeric particles, surface expression on microbial carriers, and production in biological systems that inherently protect the antigen [3,27,28,29,33,38,40,47,48,49,51,67,68]. Plant-derived platforms offer a unique advantage in that seed tissues naturally encapsulate proteins within carbohydrate- and protein-dense matrices that limit enzymatic access and slow degradation, allowing more intact antigen to reach the small intestine [4,29,36,38,39,40,41,69].

Another critical challenge is oral tolerance [69]. Repeated exposure to low or unprotected levels of antigen can prime the gut immune system to down-regulate responses rather than stimulate them, resulting in systemic irresponsiveness [29]. To avoid this outcome, successful oral vaccine platforms must deliver antigen at sufficient local concentration and in a form that promotes uptake by immunogenic antigen-presenting cells. Microbial platforms, such as recombinant yeast [49], provide inherent immunostimulatory properties through β-glucans and cell wall components, while seed-based platforms can deliver antigens in concentrated, bioencapsulated particles that achieve higher local doses. In some cases, mucosal adjuvants or co-delivery strategies further reinforce immunogenic signaling and counteract tolerogenic pathways [17].

Together, these mechanistic considerations highlight the need for antigen formats that can withstand gastrointestinal conditions, reach inductive sites intact, and present appropriate immunological cues. Different expression systems address these challenges in distinct ways as follows: microbial hosts may offer immunogenic cell walls, but require stabilization strategies [33,46,48,49,53], whereas plant-based platforms [3,4,36,38,39,41,45], particularly seed-derived antigens such as those produced in maize [34,70], provide natural bioencapsulation and particulate delivery. Understanding how each system interfaces with the biology of mucosal immunity is crucial for evaluating their suitability for oral HBV vaccine development.

## 4. Microbial Platforms for Oral Hepatitis B Vaccines

Microbial expression systems have played a central role in recombinant HBsAg production [46,47,48,49,53,71,72,73,74] and have been widely explored for oral vaccine development. The two primary microbial hosts—*Escherichia coli* [47] and yeast species [46,48] such as *Saccharomyces cerevisiae* and *Pichia pastoris*—offer distinct advantages in scalability, protein production, and well-established bioprocessing frameworks. Their long history of use in biotechnology provides a strong foundation for adapting these systems to oral delivery formats. The following sections provide an evaluation of various microbial and plant host platforms, focusing on their respective capacities to produce and deliver immunogenic HBsAg.

*E. coli*-based systems have a historical advantage of rapid growth, inexpensive fermentation, and exceptionally high yields of recombinant protein. These properties make *E. coli* an efficient platform for large-scale protein production, and numerous studies have demonstrated successful expression of HBsAg in bacterial hosts [22]. In addition, *E. coli* can be engineered to display antigens on the bacterial surface or produce outer membrane vesicles (OMVs) [75,76], both of which generate particulate structures that favor uptake by M cells in the gut [29,34,77,78] and can provide intrinsic immunostimulatory signals [3,4,13,51,75,76]. These features highlight the following important strengths: flexibility in antigen format, high productivity, and the potential for particulate delivery systems.

However, *E. coli* also presents challenges. HBsAg expressed in bacteria often accumulates in inclusion bodies, requiring solubilization and refolding [54,79] to restore conformational epitopes and assemble into virus-like particles (VLPs), which are crucial for immunogenicity. As a soluble recombinant protein, bacterial HBsAg requires appropriate formulation, such as encapsulation or the use of protective matrices, to maintain its structural integrity and ensure effective delivery [6]. Therefore, it is necessary to design formulations to overcome the inherent vulnerability of non-encapsulated proteins to the gastrointestinal environment. Furthermore, *E. coli* cannot perform post-translational modifications such as glycosylation, which are essential for some antigens.

Yeast-based systems offer several advantages, particularly relevant to oral vaccinations. Yeast can perform eukaryotic folding and post-translational modifications, enabling HBsAg to assemble into the native VLP [36,44,46,48,52,72,80], the antigenic form used in licensed injectable vaccines [3,13,23,38,51,68]. VLP formation can trigger an innate immune response, improve stability, and support more efficient uptake by GALT through particulate transport pathways [63,81], and increase the likelihood of inducing active immunity rather than tolerance. Beyond VLP assembly, the yeast cell wall, composed of β-glucans, mannoproteins, and chitin, provides adjuvant properties [48,82], engages pattern recognition receptors such as dectin-1 on DCs [48,69,78], and promotes immunogenic rather than tolerogenic signaling [9,29,48,51,66,69]. These innate-stimulatory properties make yeast particularly attractive for mucosal vaccine applications.

Despite these strengths, yeast platforms are not without limitations. A relatively large biomass may be required to achieve sufficient antigen dose for oral administration, as only a fraction of cells express high levels of recombinant HBsAg. Yeast-derived VLPs are essential for high immunogenicity; however, these multimeric structures are highly fragile and susceptible to disassembly or denaturation under the harsh conditions of the gastrointestinal tract, such as low pH and proteolytic activity. Therefore, sophisticated formulation strategies are required not only to protect the protein itself but specifically to preserve the structural integrity of the VLPs, ensuring effective immune recognition.

Taken together, microbial platforms offer the following clear advantages for oral vaccine development: high production capacity, established safety profiles, multiple antigen-presentation formats, and inherent immunostimulatory properties. At the same time, challenges involving gastrointestinal stability, antigen folding, and dose standardization highlight areas where additional engineering or formulation strategies are needed. These strengths and limitations provide an important benchmark for evaluating alternative platforms.

## 5. Plant-Based Platforms for Oral Hepatitis B Vaccines

Plant-linked production systems for oral HBsAg include unicellular chassis (such as algae and plant cell suspensions) and whole-tissue crops (like tubers, leaves, and seeds) [52]. A platform is suitable for oral vaccination only if it ensures (1) correct antigen structure—ideally VLP-like, disulfide-matured HbsAg [3,44,51]; (2) matrix-mediated protection against gastric acidity and proteases to reach GALT [17,29,39,51]; and (3) dose standardization to subcellular trafficking to prevent low-dose tolerogenic exposure [29,51,69]. These requirements depend on the Endoplasmic Reticulum (ER)/Golgi/vacuoles es (PSVs) or plastids, tissue protease environments (such as vacuolar processing enzymes in leaves and fruit), storage organelle properties (protein bodies or oil bodies), encapsulation (e.g., within cellulosic cell walls, starch/protein matrices or lipid droplets), and mucosal immunobiology (including M-cell uptake, DC programming, and regulatory T cell (Treg) induction). At the same time, these systems must possess the practical advantages of producing a cost-efficient, scalable, and ideally thermostable product.

## 6. Algal Systems (e.g., Chlamydomonas)

Unicellular green microalgae (e.g., Chlamydomonas reinhardtii) offer contained, photobioreactor-based production and mass-based dosing as biomass or dried powders, giving a particulate delivery format that can be standardized and stored at ambient temperature [3,38]. Most molecular designs place transgenes in the chloroplast genome, leveraging plastome polyploidy for high copy number and facilitating homologous recombination at defined loci; chloroplast accumulation also minimizes transgene silencing and supports high yields of recombinant proteins in a compartment that naturally assembles complex macromolecular machines [38,71,72,74]. These attributes favor particulate oral delivery (powders/capsules) and stability under distribution constraints.

Chloroplast cannot perform N-glycosylation [3,38,52], which is irrelevant for some antigens but can influence membrane protein conformation and VLP assembly; hence for HBsAg, a cysteine-rich lipid-associated protein whose neutralizing epitopes depend on disulfide-stabilized quaternary organization, formulation must promote conformational stability and particle-like presentation. Dried algal powders can protect antigens from hydrolysis and acid, preserving uptake by GALT, and algal chloroplasts have historically supported immunogenic vaccine candidates and GMP-amenable processes [3,38,52,74]; nonetheless, for HBsAg specifically, published evidence is largely at the bioprocess/feasibility level, and human data are lacking. Typical reported algal antigen accumulation (8–21 mg/g dry weight [38,74]) is compatible with oral formats but remains variable, and regulatory pathways for microalgal oral vaccines (dosage uniformity, environmental containment, and allergenicity) are still maturing.

Accordingly, algae provide a contained, particulate, shelf-stable material aligned with oral dosing logistics and GALT sampling, but for HBsAg, they lack glycosylation, clinical experience, and a seed-like bioencapsulation record needed for near-term translation.

## 7. Plant-Cell System (Tobacco BY-2, Carrot Cells)

Plant-cell suspensions (e.g., tobacco BY-2, carrot cells) are sterile and have bioprocesses with controlled media and scale-up capabilities similar to microbial fermentations [83,84]. They can produce clinical-grade antigen batches and integrate with GMP [3,39]; however, since the biomass is not in a readily consumable form, the products are usually purified and formulated for oral delivery. GI protection and dose uniformity issues shift from the tissue level to pharmaceutical encapsulation and adjuvant treatment.

BY-2’s rapid cell cycle and homogeneous form support stable production and media optimization, including bioreactor-scale nutrient design and aeration control to boost productivity and biomass quality [85,86]. These tobacco cell line systems are best-suited for parenteral vaccines and are increasingly adaptable for producing complex proteins. Meanwhile, carrot cells are the most advanced toward clinical application among plant-cell suspensions, with the first FDA-approved plant-cell-derived therapeutic produced in this system [4]. While HBsAg-specific oral clinical trials are lacking for this platform, the feasibility of plant-cell manufacturing has been demonstrated through other mucosal immunization strategies and oral delivery testing using different antigens [3]. Both BY-2 and carrot suspensions lack an oral storage matrix in their raw form, thus HBsAg VLPs must be protected after purification (e.g., with acid-stable coatings or microparticles). Additionally, standardization of the dose must be achieved through conventional fill-finish processes.

Contained plant-cell platforms provide processing control and regulatory familiarity. However, their economic viability is often challenged by slow cell doubling times and lower biomass throughput compared to mammalian or microbial systems. These systems are also at a relatively high cost compared to other plant-produced proteins. The technical difficulties, combined with a lack of an oral matrix and the need for complex purification, result in higher total production costs than mammalian cell cultures.

## 8. Tuber-Based Systems (Potato)

The limitations with plant cell cultures have largely shifted to oral delivery platforms for tubers, leaves, and seeds whose developmental physiology better suits desiccation, storage, and bioencapsulation. Potato provided the first proof-of-concept for oral plant HBsAg in a human trial, demonstrating that oral administration of plant-produced antigen can elicit mucosal (IgA) and systemic responses, a historical pivot that validated plant-produced oral vaccines [3,4,38,51,52,84].

Parenchymatic cells in tubers are water-rich and protease-active, especially cysteine proteases, which compromise post-harvest antigen stability and VLP integrity unless the antigen is sequestered away from vacuolar proteases through subcellular targeting or tissues are quickly lyophilized [51]. HBsAg levels in tubers (<0.016 mg/g FW) limit the economics and palatability of dosage per mass [3,30,51,84], and the physiology of starch storage does not naturally support membrane-protein particle assembly or acid survival [50,51]. Therefore, high intake volume and a cold chain are necessary to slow proteolysis.

While Phase I studies confirmed feasibility [30,51], tubers remain an experimental platform rather than a scalable, translational solution. Tuber biology revealed that palatability alone is not sufficient. Storage physiology and protease profiles must also support stability and dosage. Furthermore, the refractory nature of raw tuber starch (Type 2 resistant starch) hinders antigen release in the small intestine, rendering it largely nondigestible and limiting its effectiveness as an oral delivery vehicle. Unlike leafy or fruit tissues, where the cellular matrix balances protection and accessibility, the crystalline starch in tubers necessitates pre-treatment or cooking, which often compromises protein integrity. This shifted the focus of the community toward leafy and fruit tissues (palatability) and seeds (desiccation-enabled stability).

## 9. Leafy Crop Systems (Lettuce, Tomato, Tobacco Leaves)

Leafy and fleshy tissues improve palatability and allow raw consumption [3]. However, they are metabolically active, contain high-water matrices with vacuolar/lysosomal proteases [3,51] (including vacuolar processing enzymes, VPEs) and result in short shelf-lives for the antigen [37,50,51]; unless stabilized [54] (e.g., chloroplast targeting + lyophilization), soluble antigens degrade [29,55]. Furthermore, uniformity of antigen levels drifts across maturation or ripening gradients [3,51,52].

In lettuce, chloroplast transformation concentrates the antigen in plastids, supports high gene copy number, and enables dry-powder or tablet formats that withstand room temperature and provide protection in simulated gastric fluid for meaningful windows [3,30,38,39,51]. While nuclear transformation typically results in lower HBsAg (0.00006–0.060 mg/g FW), plastid designs can significantly raise antigen loads. However, they lack eukaryotic glycosylation. Mucosal adjuvants (e.g., Cholera Toxin (CT)) or carrier proteins (e.g., Cholera Toxin subunit B (CTB)) can elicit mucosal immunity at lower doses [3,36,38,39,87].

Tomato introduces ripening-linked protease activity and plastid-to-chromoplast transitions that alter proteostasis and raise pH [88,89]. These developmental changes stress VLP assembly unless sequestration or drying is applied at early post-harvest.

Tobacco leaves provide very high transient or stable yields and have supported human data for purified antigens [86,90,91] (e.g., a licensed plant-made COVID-19 vaccine in Nicotiana [92]), but because tobacco leaves are not suited for oral delivery, downstream purification and encapsulation are required. There is an abandonment of the oral-matrix benefit and it largely recapitulates the burdens of conventional formulations.

Leafy/fruit systems confirmed the following two main conclusions: (i) plastid engineering and lyophilization significantly enhance stability and room-temperature logistics; (ii) watery tissues remain susceptible to proteolysis and antigen drift.

## 10. Seed-Based Systems: Rice

Seed development programs provide desiccation tolerance [3] and storage organelles that encapsulate protein and lipid reserves for post-germination growth [51]. This physiology provides stability under ambient conditions and bioencapsulation, slowing gastrointestinal degradation, buffering against low pH, and shielding against enzymes [28,93,94,95]. Seeds can be ground into flour [3,62], which enables lot-to-lot blend-back, for dose standardization, an essential requirement for avoiding tolerogenic under-dosing and for meeting regulatory content-uniformity expectations.

In rice, HBsAg can be targeted to endosperm protein bodies [50] (PB-I (Protein Body Type I), which are prolamin bodies in rough ER, and PB-II (Protein Body Type II), which are glutelin bodies in protein storage vacuoles (PSVs). The molecular physiology of PB-I and PB-II trafficking is well-studied, such as dense-vesicle sorting via trans-Golgi network (TGN) to PSVs, and rough-ER retention for prolamins, and mutations that perturb endosperm targeting (e.g., glutelin precursor accumulation or post-Golgi mis-sorting) illustrate how sensitive storage organelle biogenesis is to secretory traffic and how easily a recombinant membrane antigen might be destabilized if mistargeted [50,96]. The antigen can benefit from protein-body sequestration and long-term ambient stability [50,51]. However, the low expression levels (~32 ng/g seed) [3,51,97] limit the dose per unit mass unless additional concentration is performed. This makes economics and palatability challenging and raises the risks of tolerance.

## 11. Seed-Based Systems: Maize

**Figure 1:**
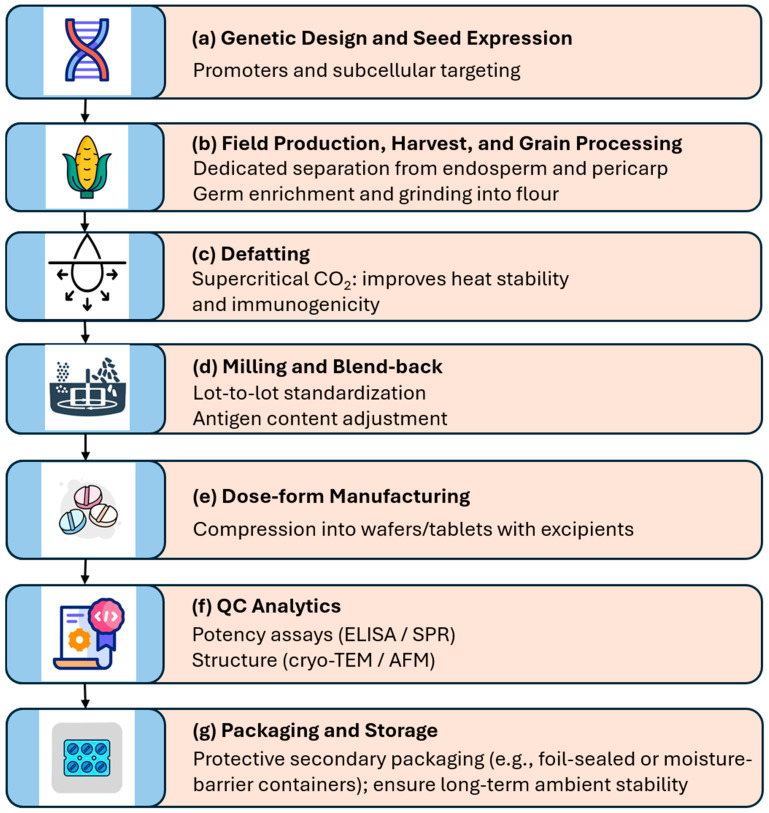
**Manufacturing and quality control workflow for maize-derived oral HBsAg vaccines.** The translational pipeline for oral vaccine production leverages the unique physiological properties of maize germ. (**a**) Genetic design: high-level expression is driven by embryo-preferred promoters, ensuring targeted accumulation within the germ matrix [98]. (**b**) Field production: scalable agronomic practices allow for large-scale antigen harvesting, followed by dedicated mechanical separation from endosperm and pericarp. (**c**) Defatting: supercritical CO_2_ extraction removes endogenous lipids, a critical step that enhances the heat stability of the bioencapsulated antigen up to 55 °C and increases immunogenicity [99,100]. (**d**) Milling and blend-back: processed germ is standardized to ensure lot-to-lot consistency in antigen content. (**e**) Dose-form manufacturing: compression into wafers or tablets with excipients provides a practical, needle-free delivery format suitable for field deployment [101]. (**f**) QC analytics: final product quality is verified through potency assays (Enzyme-Linked Immunosorbent Assay (ELISA)/Surface Plasmon Resonance (SPR)) and structural analysis (Cryogenic Transmission Electron Microscopy (cryo-TEM)/Atomic Force Microscopy (AFM)) to ensure VLP integrity and compliance with ICH Q1A/Q5C stability standards [102,103]. (**g**) Packaging: finished units are placed in moisture-barrier packaging (such as foil-sealed pouches or blister packs) to preserve the vitrified state of the matrix and ensure structural integrity during ambient storage and transport.

In addition to the inherent properties of using seeds for vaccine production, maize has characteristics that make it particularly well-suited for oral delivery. While rice utilizes protein bodies in the endosperm, the unique physiological architecture of maize, specifically the embryo or germ, provides an even more robust environment for HBsAg. Maize seeds uniquely combine (1) precise interaction with lipids to maintain antigen integrity, (2) high-level accumulation of a membrane-associated, disulfide-rich antigen, (3) matrix-driven bioencapsulation that limits acid and protease access, and (4) mill-and-blend dose standardization compatible with GMP content uniformity targets [3]. These four factors collectively outperformed watery tissues like potato, lettuce, and tomato, which required additional encapsulation for contained plant-cell systems. The translational pipeline for maize-derived HBsAg, from genetic design to final patient-ready units, is summarized in Figure 1. Each step is optimized to ensure high antigen accumulation, structural integrity, and dose uniformity.

There are a multitude of reports demonstrating the accumulation of recombinant proteins in maize seed. What makes the maize host particularly beneficial for HBsAg is the ability to target expression to the embryo (germ). Targeting HBsAg to the germ leverages the unique biology of oil bodies (lipid droplets), which consist of a triacylglycerol (TAG) core wrapped by a phospholipid monolayer and stabilized by proteins such as oleosins, caleosins, and steroleosins [104]. The TAG-to-oleosin ratio is critical, as it influences droplet size, coalescence resistance, and biophysical stability under desiccation and heat [105]. This lipid-rich, semi-hydrophobic microenvironment physically shields amphipathic viral membrane proteins, such as HBsAg, effectively masking epitopes from proteases while slowing conformational unraveling during gastrointestinal transit [68]. Understanding this interaction between expression design and subcellular routing explains why the germ oil-body environment outperforms other seed compartments for oral vaccine stability [68,105].

While lipids are critical to maintain HBsAg integrity, they can also be oxidized, creating stability problems. HBsAg in the seed is stable, but once milled, the lipids are susceptible to oxidation. This leads to interactions with proteins that can degrade protein integrity. It was shown that without removing excess lipids, the VLPs in maize were highly aggregated. When the standard method of hexane extraction was performed, the VLPs were of a more uniform size; however, they were slightly different than that of the native VLP as seen by transmission electron microscopy (TEM) and circular dichroism (CD) [102].

Another method of removing lipids is the use of supercritical fluid extraction (SFE) with CO_2_ [99] (Figure 1c). This food-friendly process primarily removes non-polar storage lipids (TAGs) while leaving the polar phospholipids associated with HBsAg intact. The removal of bulk storage lipids increases the antigen-to-matrix ratio and reduces the potential for lipid oxidation, which can interfere with protein stability. As lipids play a critical role in stabilizing membrane-associated proteins and maintaining membrane dynamics [104], this refinement of the lipid environment, maintaining the phospholipid/oleosin shell while reducing bulk oil, likely stabilizes the epitopes and contributes to the optimal heat stability observed in maize [99].

This resulted in maintaining HBsAg integrity as seen by TEM and circular dichroism. SFE also greatly improved the stability of the antigen compared to full-fat or hexane-extracted maize germ. SFE-treated germ was stable at 45 °C for at least 1 month and stable at ambient conditions for up to 1 year [55]. Finally, the SFE treatment increased the immunogenicity of HBsAg [101]. Thus, SFE treatment is a significant advancement in overcoming many of the barriers seen with orally delivered candidates.

Structural integrity affects VLP epitopes, disulfide maturation, and thermostability [7,9]. Biophysical studies [100] indicate that the desiccation and defatting stages (specifically SFE) serve as critical windows for VLP maturation. During these stages, controlled oxidative and thermal treatments can “harden” the particles, reducing molecular flexibility and enhancing morphological regularity [54,55]. To ensure this, parameters such as moderate humidity and redox control must be optimized during processing to facilitate, rather than compromise, these structural transitions, aligning with modern strategies for protein cargo encapsulation and VLP stabilization [94].

While the qualitative aspects of HBsAg expression in the germ make this ideal for HBsAg production, it is also critical that there must be high concentrations in the germ to make this palatable and economically attractive. The architecture of maize tissue-preferred promoter activity, intracellular targeting, and subcellular processes led to a VLP-competent HBsAg (Figure 1a) [98,101]. High accumulation was achieved by using embryo-preferred promoters (such as globulin-1 derivatives) [55] to drive expression during seed maturation, when oil body formation is at its peak. The antigen moves through the ER, where disulfide bonds form and membrane insertion occurs [106], then partitions into lipid-rich microdomains that restrict access by water-based proteases [107].

Mechanical food processing equipment is available and it can separate the germ fraction from the starch-rich endosperm. This can further increase the concentration of HBsAg by an additional 7-fold. Previous reports indicated HBsAg levels at 0.5–1 mg per gram [55,99,101], and the current seed line has produced >2 mg of HBsAg per gram dried germ (unpublished data). These concentrations are orders of magnitude higher than those reported in other edible plant hosts, enabling dosing in milligram quantities with only gram quantities of the biomass, making this easily palatable. Furthermore, at this concentration of HBsAg, the cost of raw material is less than $0.01/dose.

**Figure 2:**
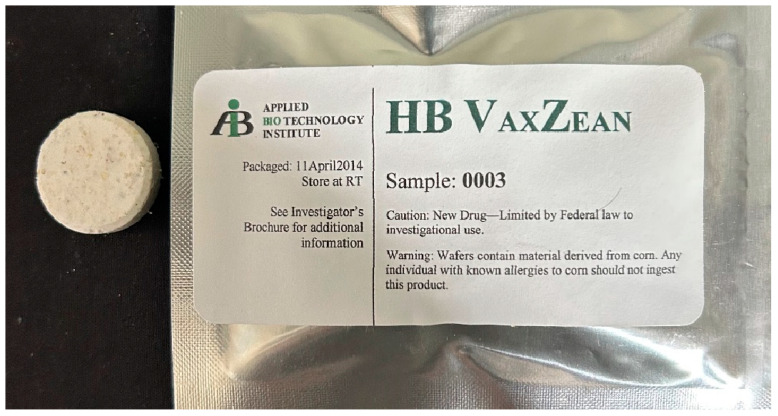
**Prototype dosage form and packaging of maize-derived hepatitis B vaccine.** Representative 2.5 cm maize-derived HBsAg wafer (HB VaxZean) displayed alongside its primary moisture-barrier aluminum foil packaging. The “Store at RT” label confirms the formulation’s stability at ambient temperatures, eliminating the need for cold-chain logistics.

After post-harvest germ enrichment and SFE treatment, the material can be ground into flour. Milling into flour enables blend-back with commodity corn for per-unit content uniformity with wafer/tablet dose forms [28]. This approach also allows for excipients to be added that can further increase stability when making tablets. The tablets can then be sealed in airtight packages for an easy-to-administer form (Figure 1g and Figure 2). Functionally, VLP integrity and disulfide maturation, both essential to neutralizing epitope display, are retained after storage and processing [3] (Figure 2).

Wafers and tablets can be produced via direct compression, ranging from 2 to 5 g each [101]. The pharmacopeial standards for content uniformity (e.g., 85–115% of label claim in accordance with USP General Chapter <905> Uniformity of Dosage [103]) are maintained through lot-to-lot blending of defatted germ meal to reach a defined HBsAg assay target (Figure 1d). This is followed by direct compression into wafers or tablets (Figure 1e), formulated to align mg/g accumulation with practical adult booster doses [101].

Their uniformity is established through detection assay techniques such as an ELISA, orthogonal liquid chromatography, or immune-potency testing, where applicable, along with content-uniformity assessments of finished units (Figure 1f). Stability protocols should incorporate accelerated conditions (e.g., 40 °C/75% RH as defined by the ICH Q1A (R2) guideline [108]) and ambient storage, complemented by structure-function correlation methods (e.g., SPR and cryo-TEM) to comply with ICH Q5C [103] for the specific requirements of the biotechnological products guideline, focusing on the maintenance of antigenic potency and VLP structural integrity.

Disintegration testing in simulated saliva, simulated gastric fluid (SGF), and intestinal fluid (SIF) can be adjusted with excipients, but primary protection mainly comes from the germ matrix and defatting state [28,99]. Limiting free water during storage prevents pre-hydration that could speed up gastric release and raise tolerance risk [69], as premature antigen exposure in the stomach often leads to immune desensitization rather than the desired systemic response.

Dose forms are only effective if they trigger the correct immune profile [101]. HBsAg immunogenicity correlates with VLP assembly and proper intra- and inter-molecular disulfide bonding. Maize germ expression provides both the folding environment and lipid context necessary for these maturation steps [104]. The ER-associated processes promote disulfide formation, while the unique lipid-body microenvironment supports the assembly of 22 nm spheroidal particles [106].

**Figure 3:**
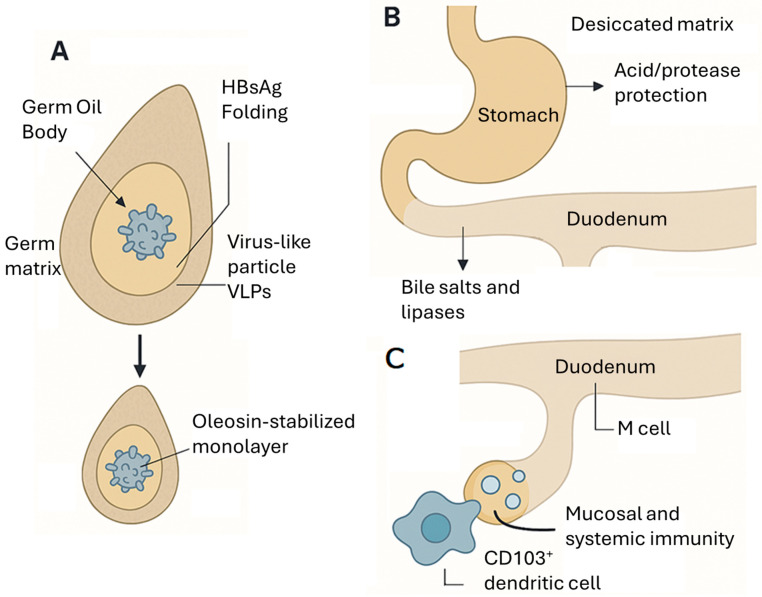
**Mechanistic overview of maize-derived HBsAg stability, release, and mucosal immune activation.** (**A**) Molecular and tissue-level stabilization: within the germ cells, HBsAg folds into virus-like particles (VLPs) and localizes to oil bodies. The oleosin-stabilized monolayer, combined with the naturally desiccated germ matrix, protects VLP structural integrity and prevents aggregation during seed maturation and storage [99,108,111]. (**B**) Gastrointestinal Survival and controlled release: following oral intake, the dry plant matrix provides a bioencapsulation shield that protects the antigen from acid and proteolytic environment of stomach [109,113]. Upon transit to the duodenum, bile salts and pancreatic lipases emulsify the residual lipids, triggering the timed release of membrane-associated HbsAg [100]. (**C**) Mucosal and systemic immune induction: released antigens are sampled by intestinal M-cells based on the general mucosal sampling pathway [78] and presented to CD103^+^ DCs within Peyer’s patches. By delivering a high local dose density in a particulate format, the system promotes immunogenic programming over tolerance, inducing both mucosal IgA and systemic IgG responses, effectively countering oral tolerance [69,101].

Seed bioencapsulation and desiccation physiology are fundamental for converting plant development into oral stability of the proteins [4,39]. The mechanistic journey of maize-derived HBsAg, from tissue stabilization to gastrointestinal release and eventual immune activation, is illustrated in Figure 3. This process ensures that the antigen remains immunogenic until it reaches the targeted lymphoid tissues. Seed physiology includes desiccation tolerance, the formation of a glassy cytoplasmic matrix (vitrification), and storage organelles. This glassy state immobilizes proteins and prevents molecular collisions, inhibiting protease activity and water-induced unfolding, offering room-temperature stability [55] that leafy or tuber tissues cannot match (Figure 3A). In maize germ, lipid bodies are embedded among protein and carbohydrate matrices, which further slow access by pepsin, trypsin, and bile salts [94]. During rehydration in the small intestine, as generally demonstrated in pant tissue models [29], gradual matrix relaxation and lipase or protease activity facilitate the release of VLP-competent HBsAg near Peyer’s patches [29,68].

Structural integrity of the antigen must withstand physiological conditions. This involves protecting it from the gastrointestinal environment and understanding the kinetics of how the antigen is released from maize germ matrices. At the immunobiology interface, successful oral vaccines must deliver intact, particulate, or membrane-associated HBsAg to GALT and mucosal inductive sites in the oral cavity while avoiding low-dose tolerogenic exposure [99]. Maize germ’s lipid-body microenvironment stabilizes the membrane antigen and supports dose-dense units. These oral formats facilitate prolonged mucosal contact, potentially activating sublingual and tonsillar lymphoid tissues, pathways known to enhance distal immune responses, including those in reproductive tissues, thus ensuring the local antigen concentration at inductive sites exceeds tolerogenic thresholds and promotes M-cell sampling and immunogenic dendritic programming [78].

The desiccated, lipid-rich germ matrix slows water ingress and pepsin access to the encapsulated protein, while the phospholipid/oleosin interface resists acid-induced interfacial denaturation, thereby delaying epitope exposure until the post-pyloric pH rise and bile salt emulsification in the duodenum [68,94,95] (Figure 3B). Although information on direct maize-HBsAg SGF and SIF kinetics are still limited in commercial vaccines, analogous plant matrices (lyophilized chloroplast tissues) protected vaccine proteins for ≥60 min in SFE and demonstrated the effect of SIF-delivered proteins [100]. Furthermore, it was demonstrated that an orally delivered recombinant protein in the maize matrix could be detected in fecal matter, while the orally delivered purified form could not [34]. These results support the general bioencapsulation principle that maize uses to protect antigens and enable higher antigen density at the target site.

Emulsification of residual lipids and lipase activity on TAGs in germ promotes gradual antigen release near Peyer’s patches, increasing the likelihood of M-cell transcytosis and presentation by CD103^+^ DCs [51] (Figure 3C). Achieving an immunogenic (non-tolerogenic) response depends on the local dose and innate cues or adjuvants. With controlled release near GALT and mg/g-level loading, the next step in translation involves dose-form design and standardization.

The immunogenicity strategy begins by evaluating preclinical signals. Decades of HBV vaccinology show that neutralizing anti-HBsAg antibodies correlates with protection [7,19]. Parenteral priming establishes memory B cells and T follicular helper (Tfh) cells help, while oral maize boosters can re-stimulate and add mucosal IgA at potential exposure sites. The parenteral-prime plus oral-boost approach explicitly avoids oral tolerance as the main induction step and is supported by the oral-vaccine literature as the most practical route for HBV [3,107].

Adequate local dose density, particulate/membrane presentation, and danger-signal context (e.g., plant cell-wall fragments, β-glucans if co-formulated) promote immunogenic DC programming [109] and inhibit Foxp3^+^ (Forkhead box P3 protein expressed in the T-cell) Treg induction by tolerogenic DCs, which are core principles of modern oral-tolerance immunology. To apply this, mucosal adjuvants, VLP assembly, ingenious antigen protection and processing, and control of high dose and timing were studied to regulate Treg induction.

Oral administration in preclinical studies elicited both a mucosal and a systemic response (Figure 3C). According to the WHO, the threshold for HBV protection can be measured using a specific anti-HBV biomarker with levels ≥ 10 mIU/mL) [101]. Maize-derived HBsAg, when orally delivered, produced geometric mean titers in serum exceeding 12,000 mIU/mL in a mouse trial [101]. The protective effects of both serum IgG and mucosal IgA exposure offer dual immunity for recipients. Additionally, the maize oral delivery booster triggers an anamnestic response. This strong response is generated by using natural maize-derived “danger signals” to bypass oral tolerance, while sustained effectiveness is maintained through rigorous manufacturing, agronomic reliability, and lot-to-lot analytics [110].

HBsAg can also be co-formulated with mucosal adjuvants to increase the immune response and counter oral tolerance (e.g., particulate presentation, β-glucan-like signals, or CT-based strategies) [17,29,39,51]. However, when the *E. coli* labile toxin was used as an adjuvant, no significant difference could be observed [55]. This was likely due to the relatively high concentration of antigen used. These studies directly address gastric survival, particle-like presentation, and per-unit dose control, the three failure points of earlier platforms.

To ensure universal protection, it is important to consider vaccine non-responders, those that fail to achieve a protective anti-HBs titer (≥10 mIU/mL) despite these natural signals. Non-responsiveness can stem from host-related factors (e.g., age, obesity, and smoking) or underlying clinical conditions (e.g., diabetes mellitus, chronic kidney disease, human immunodeficiency virus (HIV) infection, and immune suppression) [8,13,58]. Identifying these high-risk groups is essential for implementing targeted strategies to overcome more persistent oral tolerance. For these individuals, advanced approaches such as higher doses, CTB fusions [51] to enhance antigen uptake and Cytosine-phosphate-guanine Oligodeoxynucleotide (CpG ODN) entrapment [27] to promote immunogenic DC programming and inhibit Foxp3^+^ Treg induction represent excellent options to ensure broader vaccine coverage and effective seroconversion. It has also been shown that co-administration of a parenteral vaccine and the maize-produced oral vaccine candidate can be synergistic, leading to either reducing the number of boosters required or a stronger immune response in non-responders.

Alongside these immunological considerations, manufacturing and agronomic aspects such as scale, genetics, containment, and QA/QC are crucial for the large-scale development of the platform. Maize’s extensive global acreage, hybrid seed systems [98], and trait fixation facilitate dedicated production blocks and appropriate isolation distances. To address regulatory and safety concerns, rigorous identity preservation (IP) systems, including contract growing and dedicated milling equipment, are used to prevent the commingling of food corn and ensure compliance with USDA regulations (Figure 1b). To ensure consistent dose economics, trait stability should be monitored through kernel-lot ELISA, qPCR, and expression audits across seasons, ensuring stable mg/g outputs [111].

In-process controls include HBsAg potency assays (ELISA, epitope mapping), particle assays (cryo-TEM/AFM [31,111] where feasible), residual oil content testing (to monitor SFE efficiency), and rigorous testing for microbial and chemical purity on finished oral units. For VLPs, non-intrusive structural assays validated on licensed rHBsAg [31,111] can be applied to maize lots for comparability. Since this is a genetically modified (GM) oral vaccine, the regulatory strategy must incorporate comprehensive safety and quality data into a formal dossier.

Beyond process controls, regulatory considerations for GM-oral biologics involve environmental assessments, covering gene flow management and volunteer control, as well as stringent food-grade safety measures addressing mycotoxins, heavy metals, bioburden, and contaminants [20]. Ensuring biological quality involves verifying identity, potency, uniformity, and stability. Additionally, clinical trial design must be aligned to demonstrate oral efficacy by demonstrating the titer of the WHO biomarker. The oral-matrix advantage of maize shifts the Chemistry, Manufacturing, and Control (CMC) focus from purification to controlling in-matrix release. While a regulatory pathway to first-in-human exists, there are gaps in analytical, mechanistic, and clinical domains that are unique to this approach.

Future studies should examine the gastrointestinal transit kinetics in maize matrices by establishing standardized protocols for simulated gastric and intestinal fluids (SGF→SIF [112]) using defatted germ wafers [99], with a focus on time-resolved epitope retention and VLP morphology, building on existing chloroplast lyophilizate models.

Furthermore, research should focus on correlating potency by combining SPR epitope mapping [100] and cryo-TEM analyses with in vivo boosting results. This will help define the structure-function thresholds needed for lot release. Investigating adjuvant strategies may be helpful. Testing β-glucan-bearing particles or yeast-derived envelopes, mixed at low ratios, could enhance dectin-1 signaling and potentially lower tolerance risks.

Additionally, the shelf-life of maize-derived HBsAg has been characterized using Arrhenius-type kinetics [32,108] to analyze epitope decay in defatted wafers at temperatures of 25, 40, and 55 °C. These studies confirm storage stability under various climate conditions, showing that the matrix maintains antigenicity even at elevated temperatures. Furthermore, clinical sequencing studies are being utilized to determine the optimal timing between the parenteral prime and maize-oral boost intervals, such as 2–6 months, and to refine dose titration based on wafer mass. These steps will help optimize IgG titers and mucosal IgA levels, supporting the consensus on HBV oral vaccines.

## 12. Comparison of Platforms

Plant tissues vary significantly in their ability to preserve antigen structure, facilitate practical dose creation, and withstand gastrointestinal breakdown. While microbial, algal, tuber, and leafy systems each provide valuable mechanistic insights, none can simultaneously offer high antigen density, stability at ambient temperature, and straightforward dose standardization, critical factors for the success of an orally delivered HBsAg booster. These limitations naturally refocus attention on seed-based systems, where desiccation physiology and storage-organ biochemistry provide inherent stabilization advantages. Maize grain has the molecular, biochemical, and processing features that make it a practical host for developing an oral HBsAg vaccine. Maize has become the most advanced and scalable platform, capable of accumulating milligrams of antigen per gram and providing strong bioencapsulation. Maize germ-targeted HBsAg production leverages seed desiccation physiology and oil-body biochemistry to achieve efficient production of VLP. High levels of HBsAg (>1 mg/g) have been obtained to make this palatable and cost-efficient. Bioencapsulation allows for GI survival. SFE provides high thermal stability (≥55 °C post-defatting) while increasing immunogenicity. Finally, the practical dose-form standardization and tablet formulation enable the ideal oral delivery package. These properties address the historical failure points [3,39,101], low dose, poor stability, poor immunogenicity, as well as the fears of tolerance rise, which have limited earlier oral platforms. The key characteristics, advantages, and limitations of all the discussed platforms are comparatively summarized in Table 1. To contextualize these advancements, Table 2 provides a comparative overview of the key attributes of the maize-derived oral candidate versus the current injectable recombinant standard.

## 13. Discussions

The comparative evaluation of host platforms for oral delivery of HBsAg highlights the distinct translational advantages of maize as a host system. Early plant-produced oral vaccine systems, especially potato and lettuce, demonstrated proof of concept for oral vaccine delivery [39,62]; however, these methods were limited by low antigen yields and the large amounts needed for effective dosing. Although rice provides improved protein stability thanks to seed-based storage properties, its moderate expression levels [50] limit the practicality of effective oral administration. Tobacco systems support substantial antigen production but require purification [109], which diminishes the main benefit of direct oral consumption.

In contrast, maize can reach gram-per-kilogram levels [3] of antigen accumulation, enabling oral dosing through small, palatable units with milligrams of antigen per wafer. The seed’s natural matrix acts as a bioencapsulation mechanism [28,112], effectively protecting HBsAg from gastric degradation. Additional steps, like germ enrichment and defatting [99], further increase the antigen concentration and help standardize and stabilize the antigen. When combined with scalable agronomic practices, extended shelf-life, and heat tolerance, maize stands out as an ideal candidate for practical oral vaccine deployment.

While injectable recombinant HBsAg vaccines remain the gold standard for primary immunization, maize-based oral vaccines should be considered complementary tools within vaccination strategies. They are especially useful for booster doses, catch-up immunizations, and in field-deployable formats in low-resource settings [3,8] where cold chain logistics, medical needles, or trained personnel are limited. The success of maize-derived vaccines depends on the reproducibility of processing protocols to ensure consistent antigen content, in vitro validation of gastrointestinal stability, and preclinical studies that clarify both systemic and mucosal immunogenicity. Additionally, regulatory pathways for plant-produced orally delivered GM vaccines need to be clarified, including safety evaluations, environmental risk assessments, and batch-to-batch consistency checks.

Unlike early oral platforms that required unreasonably large amounts to be consumed, maize seeds, especially the germ, contain high levels of antigens within a naturally protective matrix that supports bioencapsulation, heat stability, and long-term storage. Processing methods, such as germ defatting and converting into wafers or tablets, concentrate the antigen and enable precise, small-dose packaging, making mass vaccination campaigns more feasible.

Importantly, maize-based oral formulations preserve the following two key benefits of the oral route: needle-free delivery and elimination of cold-chain logistics. The thermal resilience of maize-derived HBsAg is a defining advantage. Characterization by Shah et al. [100] demonstrated that while liquid recombinant vaccines are susceptible to rapid aggregation, the bioencapsulated form within the maize germ matrix maintains its antigenic profile and VLP structural integrity even under significant thermal stress. Notably, following supercritical fluid defatting [99], the antigen remains stable at temperatures as high as 45 °C [100]. This stability, combined with low water activity in the seed architecture, supports a robust shelf-life that aligns with ICH Q1A [102] and Q5C [105] standards, making mass vaccination campaigns in resource-limited settings far more feasible.

Nevertheless, it is important to recognize that oral and injectable vaccines serve distinct biological and programmatic functions and should be viewed as complementary rather than competing strategies (Table 2). Injectable recombinant HBsAg vaccines are the proven standard for primary immunization, supported by well-established correlates of protection. In this context, maize-based oral vaccines are ideally suited as boosters [107]. The unique lipid-body microenvironment and seed vitrification ensure that not only the HBsAg protein but also its crucial VLP quaternary structure remains intact during processing and gastric transit. Furthermore, the ability to trigger immunity at oral inductive sites may enhance distal mucosal protection, including the reproductive tissues. Importantly, this robust mucosal T-cell stimulation also implies potential future avenues for therapeutic vaccination to reactivate dysfunctional cellular immunity in chronically infected HBV patients [114] though its practical efficacy in chronic carriers remains to be fully evaluated in clinical settings. Nevertheless, recent strategic advances in therapeutic vaccines continue to hold significant promise for the ultimate cure of chronic hepatitis B [115]. By combining robust thermal stability (up to 45 °C) with exceptional dose economics (estimated raw material costs below $0.01 per dose), maize offers a scalable solution in resource-limited settings where conventional vaccination remains a challenge. These boosters can also be co-administered with parenteral vaccines to elicit a synergistic response, potentially aiding non-responders or reducing the total number of required injections.

To fully realize this potential of maize-based oral vaccines, future efforts must focus on ensuring consistent antigen content after processing, validating release under simulated gastrointestinal conditions [113], and building a robust body of clinical evidence in humans. Additionally, regulatory frameworks for food-derived biologic require [102] further clarification, with a focus on quality control standards, contaminant management, and environmental safety protocols for genetically modified crops. Ultimately, maize-based oral boosters could significantly enhance global adult HBV immunity, especially in populations currently missed by injectable vaccination programs [54,116]. Therefore, this platform serves as a complementary strategy to co-exist with traditional injectables.

## 14. Conclusions

There are many reports of plant-produced hepatitis B vaccine candidate using different host plants. This includes reports of oral delivery in animals and humans to elicit the appropriate immune response. Maize grain, however, appears to be the only host that can overcome the practical barriers limiting commercialization.

With success in human trials, the maize candidate could overcome many of the hurdles preventing immunization today and greatly reduce infections and fatalities that have continued after four decades of the parental vaccines. It is also anticipated that successful implementation of the orally delivered hepatitis B vaccine will catalyze the development of many other orally delivered vaccines. This approach can complement existing methods of vaccination and used as a new tool to combat disease.

## Figures and Tables

**Table 1:** **Summary of expression platforms for oral hepatitis B vaccine delivery.** Typical yields and development statuses are summarized based on representative studies. Yields are expressed as either mg/liter of culture or mg/gram of dry/fresh weight of tissue, as indicated. Reference numbers correspond to the bibliography. Abbreviations: GRAS, generally recognized as safe; GM, genetically modified; wt, weight. Data for maize germ reflect high-expression lines developed using embryo-preferred promoters [112].

Host Organism	Typical Yield	Oral Delivery Feasibility	Development Status	Key Advantages	Key Limitations
**Bacteria (e.g., Lactobacillus)**	0.1–1 mg/L [47,53,54]	Potential live mucosal vaccine	Preclinical	GRAS status; mucosal immune stimulation	Not glycosylated. Variable colonization; risk of horizontal gene transfer; strict regulatory hurdles for live GMOs
**Yeast (e.g.,** **Saccharomyces)**	0.3–150 mg/L [71,72]	Not stable when orally delivered without formulation	Gold standard for injectable vaccines	Established large-scale production; approved	Structural fragility of VLPs in SGF (disassembly); requires encapsulation; industrial processing and higher costs
**Algae (Porphyridium purpureum, others)**	8–21 mg/g dry wt [38,74]	Oral (powder, capsules)	Animal studies only	Low-cost photobioreactors; stable dried formulations; contained cultivation	Limited scalability data; lack of human immunogenicity evidence; complex cell wall hurdles
**Plant-cell suspensions (carrot, tobacco)**	25 ng–1 mg/g dry wt [83,84,109]	Oral delivery for antigens other than HBsAg	Expression verified	Contained, sterile production; regulatory familiarity	High production cost (slow doubling time); VLP structural fragility (disassembly) in SGF; requires complex purification and cold storage
**Potato**	<16 µg/g fresh wt [3,30,51,84]	Human trials showed mucosal + systemic responses	Phase I human tested [106,107]	Proof-of-principle success; easy to engineer	Impractical biomass for dosing; low digestibility of raw starch (limiting antigen release); thermal instability (antigen loss during heating)
**Lettuce/tomato**	0.06–20 µg/g fresh wt [3,4,7,36,39]	Feasible as oral tissue	Preclinical tomato Human trial lettuce [113]	Palatable, no cooking needed	High perishability; inconsistent dose content in water-rich tissues; microbial safety risks
**Tobacco (leaves)**	0.02–295 µg/g fresh wt [3,36,84,90]	Not directly applicable for oral delivery	Preclinical, some human trials with purified antigen	High yields; scalable bioreactors	Non-oral (toxic alkaloids); extensive purification required; high processing loss
**Rice (endosperm)**	<0.032 µg/g seed dry wt [51,55,97]	Human trial for antigens other than HBsAg	Preclinical	Long-term storage stability; mild processing OK	Low yield; limited enteric release data
**Maize (germ)**	<2 mg/g germ dry wt [3,28,38,51,55,99,100]	Oral; can be processed into wafers/tablets	Studies in mice are indicative of protection. Clinical trial in design, scaling feasible, extremely low cost	Ultra-high yield (mg/g level); bioencapsulation-driven SGF survival; robust thermal stability (up to 55 °C) post-defatting [99,100]; compatible with SFE for lipoid-optimized stability and purity	Regulatory barriers for GM food-crops; requires standardized dose-formulation (wafers/tablets)

**Table 2:** Key attributes of plant-based oral HBsAg vs. standard intramuscular injection platforms. This table highlights the distinct advantages of the maize-produced oral vaccine candidate in comparison to the current injectable standard. Key differentiators include the ability to induce mucosal IgA (essential for blocking HBV at entry points), superior thermostability, which eliminates cold-chain requirements, and a significantly lower cost-of-goods—critical factors for enhancing vaccine coverage in resource-limited settings.

	Maize-Produced Oral Vaccine (Candidate)	Current Injectable Vaccine (Recombinant)
**Administration**	Oral (e.g., as oral wafers/tablets) [55,101]	Intramuscular injection
**Immune Response**	Induces both mucosal (IgA) and systemic (IgG) immunity [101] (VLP-like structure is preserved)	Primarily induces systemic (IgG); weak or no mucosal response [39]
**Storage and Transport**	Thermostable; can be stored at room temperature (up to 45 °C for 1 month) [55,101]	Requires a strict cold chain (refrigeration) [13,39]
**Production Cost**	Very low; estimated raw material cost less than $0.01 per dose [28]	Higher; requires complex yeast/cell culture and purification [39]
**Ease of Use**	No needles or medical training required; eliminates needle-stick risk [55,101]	Requires trained medical personnel and sterile needles/syringes
**Current Status**	Pre-clinical testing for HBsAg in animals, indicative of protection. Feasibility of using maize platform demonstrated in human trial [55,101]	Approved and widely used globally for decades [12]

## Data Availability

No new data datasets were generated or analyzed in this study.
